# Egg oil from *Portunus trituberculatus* alleviated obesity and regulated gut microbiota in mice

**DOI:** 10.1038/s41598-020-65199-3

**Published:** 2020-05-21

**Authors:** Shiwei Hu, Huicheng Yang, Xiang Gao, Shijie Li, Wei Jiang, Yu Liu

**Affiliations:** 1 Innovation Application Institute, Zhejiang Ocean University, Zhoushan, Zhoushan 316022 China; 2 Zhejiang Marine Development Research Institute, Zhoushan, 316021 China; 3 College of Food Science, Qingdao University, Qingdao, 266071 China

**Keywords:** Obesity, Epidemiology

## Abstract

Egg oil from *Portunus trituberculatus* (*Pt*-egg oil) can overcome insulin resistance resulting from abundant bioactive lipids. However, its effects on obesity and gut microbiota were unclear. Here, we evaluated whether *Pt*-egg oil could improve obesity and gut microbiota or not in high-fat diet feeding mice. Results exhibited that *Pt*-egg oil markedly reduced body weight and adipose weight gain, improved lipid accumulation and circulatory cytokines, inhibited epididymal adipose cell size. Moreover, *Pt*-egg oil modified gut microbiota, involving decreases in the ratio of Firmicutes to Bacteroidetes, Proteobacteria, Actinobacteria, and increase in Verrucomicrobia phylum. *Pt*-egg oil reduced serum and fecal lipopolysaccharide (LPS) levels and down-regulated Toll-like receptor 4 pathway in both epididymal adipose and liver tissues. Meanwhile, *Pt*-egg oil increased short chain fatty acids and up-regulated of G-protein-coupled receptors in both epididymal adipose and liver tissues. These suggest that *Pt*-egg oil could be alternative food supplement for the prophylactic effects on anti-obesity and improvement in human gut health.

## Introduction

Obesity is a serious public health problem resulting from the high incidence. It was reported that more than 1.9 billion adults were over weight, of which the obese people were over 650 million in 2016^1^. The excess accumulation of body fat mass in obese people leads to numerous health problems and also increases the risks of series of disorders, such as type 2 diabetes, insulin resistance, hyperlipemia, and even cancer^2^. Therefore, how to prevent obesity has become a major challenge for modern societies. In the complex of factors to obesity development, gut microbiota is implicated as a master factor of nutrients uptake, energy metabolism, chronic inflammation, and other metabolic disorders^3^. Over the past decades, more and more researches confirm that gut microbiota contributes towards host metabolic homeostasis. Once the gut microbiota homeostasis is changed, many physiological disorders occur, including obesity and its complications^4^. For example, germ-free mice do not increase significantly in body weight even feeding high-fat diet (HFD), but showed remarkabe body weight gain when treated with fecal transplantation form obese animals^5^. It is reported that obesity is related with a high ratio of Firmicutes to Bacteroidetes (F/B) at the Phylum level^6^, but other paper showed that obesity is not significant related with the Firmicutes and Bacteroidetes^7^, implying that other factors may affect obesity. Perennial dysbacteriosis directly cause the physiological diseases by such secondary metabolites, especially lipopolysaccharide (LPS) and short chain fatty acids (SCFAs)^8^. As the primum movens, LPS can bind to Toll-like receptor 4 (TLR4) and subsequently activate its downstream gene, CD14^9^. LPS/CD14 pathway lowers insulin sensitivity and promotes obesity^10^. Withal, SCFAs regulate metabolic pathways through binding and activating orphan G protein-coupled receptors (GPRs)^11^. Therefore, gut microbiota directly administers obesity and its related disorders.

*Portunus trituberculatus* is one kind of swimming crab, which is widely distributed in the Western Pacific coast. Profiting from its high nutritional value and great productions, *Portunus trituberculatus* has been a significant economic marine product, more than 600,000 tons in China in 2017^12^. Current studies of *Portunus trituberculatus* are mainly involved in gene sequence analysis or aquiculture, but little papers involved in processing or utilization^13^. Our current study revealed that egg oil isolated from *Portunus trituberculatus* (*Pt*-egg oil) contained abundant phospholipids and triglyceride, which combined with a mass of eicosapentaenoic acid and docosahexaenoic acid^14^. *Pt*-egg oil remarkably reduced blood glucose and serum insulin levels in HFD-fed mice^14^. However, it is still unclear the influences of *Pt*-egg oil on obesity and the regulation of gut bacteria. Here, we investigated the effects of *Pt*-egg oil on anti-obesity and gut microbiota in HFD-fed mice, and clarify its mechanism on alleviation of obesity through regulation of microbial community and secondary metabolites.

## Results

### *Pt*-egg oil alleviated obesity

**Figure 1:**
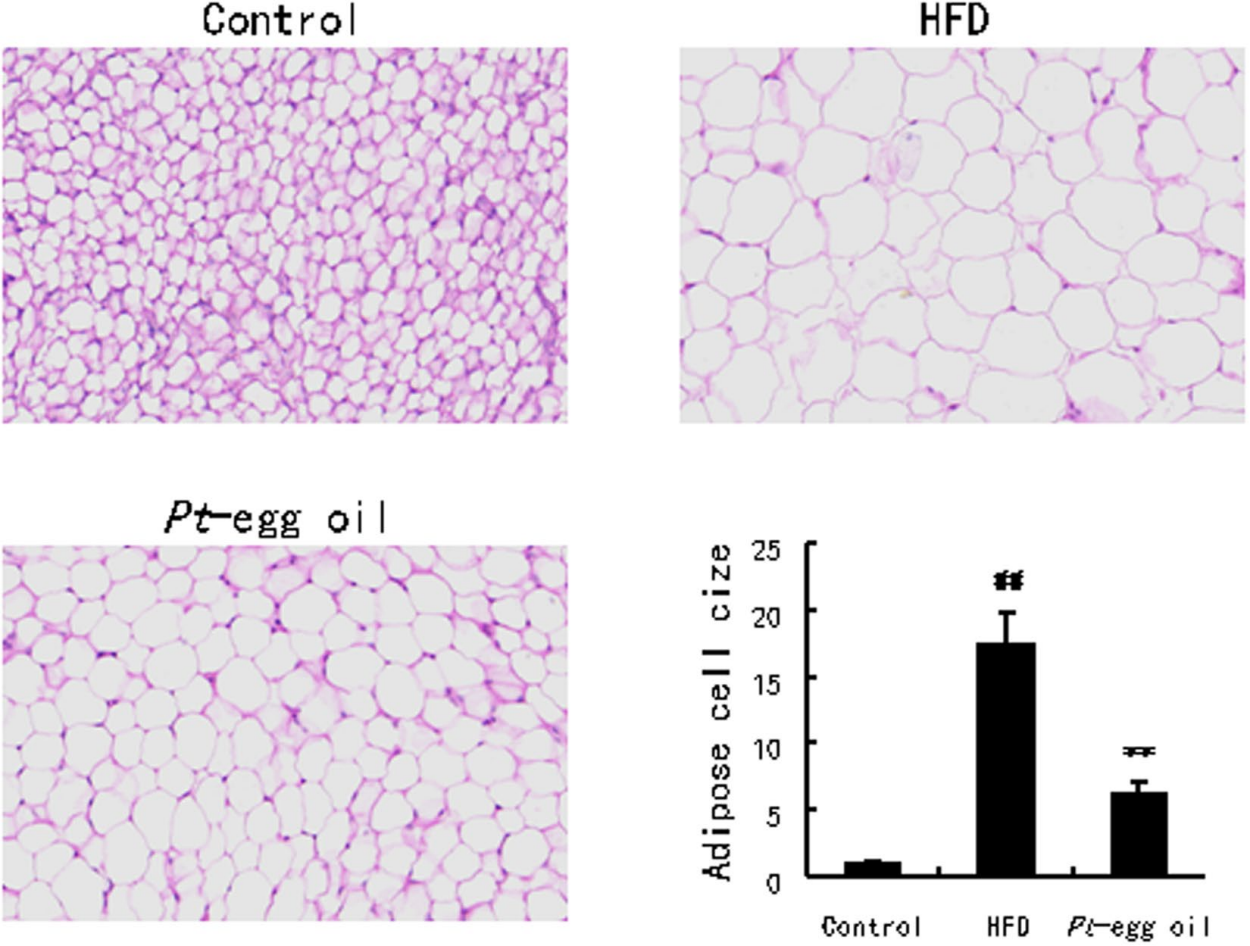
Effects of *Pt*-egg oil on the histology of adipose and liver tissues using H&E staining (*n* = 10). The adipose cell size and hepatic lipids area were measured by CaseViewer 2.0, and the size or area of the control was defined as 1. ^##^*P* < 0.01 *vs* control; ^**^P < 0.01 *vs* HFD.

Referring to our previous study, at the last experiment of feeding, the mice showed a 27.68% decrease in body weight gain in *Pt*-egg oil mice compared with that in HFD group. However, though the food intake in each mouse is much lower in HFD-feeding mice than control animals, there were significant increases in energy intake per unit weight per week in HFD, and *Pt*-egg oil groups compared with that in control group (*P*  <  0.05). As compared with the control group, the HFD group displayed an evidently higher adipose weight (*P*  <  0.01), including perirenal adipose, epididymal adipose, and abdominal subcutaneous adipose. After 16 weeks of treatment with *Pt*-egg oil, the three aforementioned adipose weights were all significantly decreased in HFD-feeding mice (*P*  <  0.05, *P*  <  0.01). In addition, Fig. 1A,B showed that HFD caused remarkable increase in epididymal adipose cell size (*P*  <  0.01). When treated with *Pt*-egg oil, dramatic decrease in epididymal adipose cell size was observed in HFD-feeding animals (*P*  <  0.01). These results indicate that *Pt*-egg oil exhibits marked antiobese effect.

### *Pt*-egg oil reduced hepatic lipids

As shown in Table 1, compared with control group, hepatic weight was significantly elevated in HFD-induced obese mice (*P*  <  0.01). *Pt*-egg oil markedly inhibited the elevation of hepatic weight by 28.34%. These changes were accompanied with the significant decreases in TG content in the liver of *Pt*-egg oil-treated mice (*P*  <  0.01), through there was no significant difference in hepatic TC content between HFD mice and *Pt*-egg oil animals. These indicate that *Pt*-egg oil can significantly decrease lipids accumulation in the liver of obese mice.

**Table 1:** Effect of *Pt*-egg oil on body or serum parameters in HFD mice.

	Control	HFD	*Pt*-egg oil
Body weight gain (g)	11.46 ± 0.82	23.99 ± 1.26^##^	17.35 ± 0.87^**^
Food intake (g/w)	28.13 ± 1.55	23.18 ± 1.74^#^	22.72 ± 1.81^#^
Energy intake (kcal/w)	115.1 ± 5.7	152.3 ± 8.1^#^	149.3 ± 8.6^#^
Perirenal adipose weight (g)	0.17 ± 0.01	0.51 ± 0.05^##^	0.31 ± 0.03^**^
Epididymal adipose weight (g)	0.54 ± 0.07	1.92 ± 0.14^##^	1.10 ± 0.19^**^
Abdominal subcutaneous adipose weight (g)	0.17 ± 0.01	0.83 ± 0.10^##^	0.59 ± 0.07^*^
Hepatic weight (g)	1.09 ± 0.10	1.87 ± 0.16^#^	1.34 ± 0.18^*^
Serum TC (mmol/L)	2.54 ± 0.16	4.56 ± 0.37^##^	3.38 ± 0.14^*^
Serum TG (mmol/L)	0.80 ± 0.07	1.35 ± 0.19^##^	0.82 ± 0.09^**^
Serum HDL-c (mmol/L)	3.41 ± 0.30	2.57 ± 0.24^##^	3.66 ± 0.34^**^
Serum LDL-c (mmol/L)	0.56 ± 0.06	1.73 ± 0.09^##^	0.95 ± 0.08^**^
Hepatic TC (mg/g)	3.24 ± 0.28	4.05 ± 0.27^#^	3.77 ± 0.12
Hepatic TG (mg/g)	22.84 ± 1.93	35.40 ± 2.46^##^	19.06 ± 1.88^**^
Serum leptin (ng/mL)	0.20 ± 0.03	0.37 ± 0.03^##^	0.22 ± 0.02^**^
Serum adiponectin (µg/mL)	0.23 ± 0.01	0.13 ± 0.01^##^	0.19 ± 0.01^*^
Serum resistin (ng/mL)	5.96 ± 0.47	12.65 ± 1.06^##^	7.83 ± 0.64^**^
Serum TNF-α (pg/mL)	33.91 ± 2.38	70.10 ± 3.77^##^	47.31 ± 3.81^**^

Data are shown as mean ± S.D (*n* = 10/group). Multiple comparisons were done using one way ANOVA. ^#^*P* < 0.05, ^##^*P* < 0.01 *vs* control; ^*^P < 0.05, ^**^P < 0.01 *vs* HFD.

### *Pt*-egg oil inhibited hyperlipemia

Obesity individual is observed with the abnormal serum lipids levels. *Pt*-egg oil treatment remarkably reduced serum TC and TG levels in obese mice (*P*  <  0.01). Moreover, serum HDL-c was remarkably increased in *Pt*-egg oil group compared with HFD group (*P*  <  0.01), while LDL-c decreased (*P*  <  0.01). These indicate that *Pt*-egg oil can significantly inhibit hyperlipemia in obese mice.

### *Pt*-egg oil regulated serum cytokines

Cytokines are closely related to obesity, such as resistin, leptin, adiponectin, and TNF-α. The data in these experiments showed that HFD obviously increased serum leptin, resistin, and TNF-α levels in mice (*P*  <  0.01), and reduced serum adiponectin level (*P*  <  0.01). When treated with *Pt*-egg oil, obese mice exhibited 40.54%, 38.10%, and 32.51% decreases in serum leptin, resistin, and TNF-α levels, and 46.15% increase in serum adiponectin level, respectively. These indicate that *Pt*-egg oil can regulate circulatory cytokines.

### *Pt*-egg oil restored gut microbiota dysbiosis

**Figure 2:**
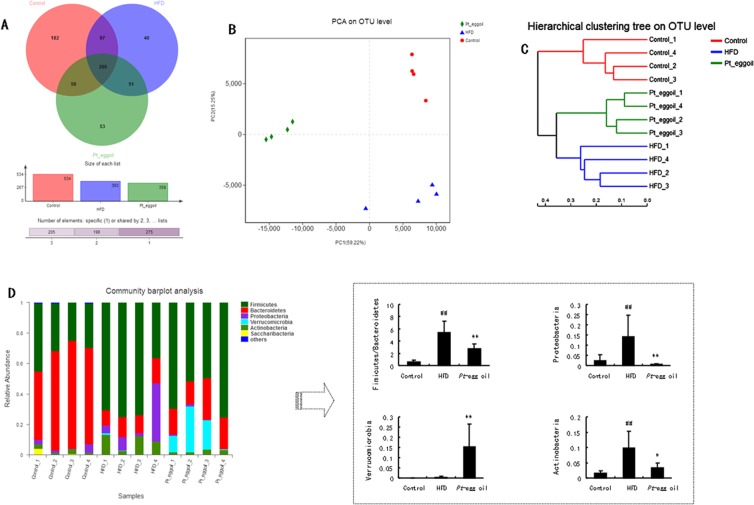
Effects of *Pt*-egg oil on the composition of the gut microbiota in HFD mice (*n* = 4). (**A**) Venn diagrams showing the unique and shared OTUs in the gut microbiota among three groups; (**B**) the weighted version of UniFrac-based Principal Component Analysis (PCA); (**C**) multivariate analysis of variance from matrix scores; (**D**) bacteria taxonomic profiling in the phylum level of intestinal bacteria from different groups. Univariate differential abundance of OTUs at the Phylum level was tested by incorporating Fisher’s exact test and the false discovery rate (FDR) among control, HFD, and *Pt*-egg oil groups and between mouse genotypes. *P* values were corrected with the Benjamini-Hochberg method to correct for the false discovery rate across multiple comparisons, which were generated using Metastats and considered significance at *P*  <  0.05. ^##^*P* < 0.01 *vs* control; ^*^P < 0.05, ^**^P < 0.01 *vs* HFD.

Gut microbiota dysbiosis contributes positively to obesity. Figure 2A showed the data of Venn diagram analysis, and three groups showed the own distinct OTUs. PCA score plot showed an oberious different microbiota distribution between all experimental groups (Fig. 2B,C). Moreover, gut microbiota at the Phylum was significantly different (Fig. 2D). F/B ratio was statistically increased in HFD group compared with control group, which was significantly reduced in *Pt*-egg oil-treated animals compared with obese mice. Moreover, *Pt*-egg oil decreased Proteobacteria, while increased Verrucomicrobia. In addition, the abundance of Actinobacteria in *Pt*-egg oil-treated mice was remarkably decreased compared with obese mice.

**Figure 3:**
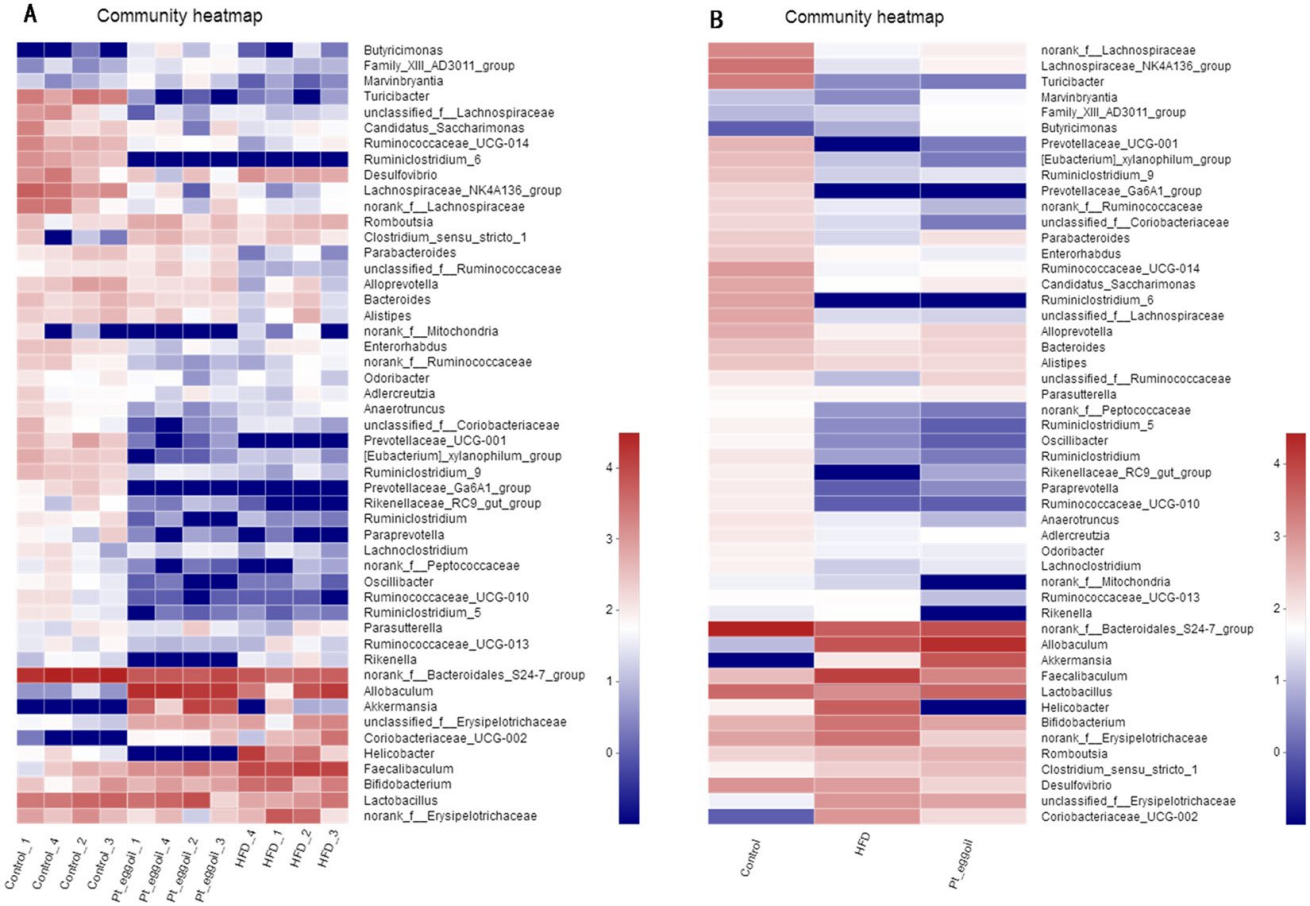
Effects of *Pt*-egg oil on gut microbiota at the genus level (*n* = 4). Heatmap indicats relative contribution of the top 50 dominat genera in each sample (**A**) and different groups (**B**). The heatmap is colour-coded based on row Z-scores.

Figure 3 showed that 48 genus exhibited remarkably different abundances in HFD mice compared with control, and 41 genus different in *Pt*-egg oil mice compared with HFD, implying that *Pt*-egg oil may alleviate obesity through regulating bacterial subset. *Pt*-egg oil reduced the numbers of *Ruminiclostridium_5*, *Ruminiclostridium*, *Ruminococcaceae_UCG-013*, *Anaerotruncus*, *Oscillibacter*, *Faecalibaculum*, *norank_f_Erysipelotrichaceae*, (all belonging to Firmicutes), *Helicobacter*, (belonging to Proteobacteria) *unclasslfied_Coriobacteriaceae* and *Coriobacteriaceae_UCG-002*, (belonging to Actinobacteria), *Bifidobactenium*, and *Desulfovibrio* compared with obese mice. While the relative abundances of *Rikenellaceae_RC9_gut_group*, *Parabacteroides*, and *Paraprevotella* (belonging to Bacteroidetes), *Lactobacillus*, *Marvinbryantia*, *Adlercreutzia*, *Candidatus_Saccharimonas*, *Family_XIII_AD3011_group*, *Asllobaculum*, and *Romboutsia* were increased in *Pt*-egg oil mice. In addition, the abundances of the SCFAs-producing microbiota *Lachnospiraceae_NK4A136_group*, *norank_f_Lachnospiraceae*, *Ruminiclostridium_9*, *Prevotellaceae_UCG-001*, *Butyricimonas*, *Alloprevotella*, *Clostridium_sensu_stricto_1*, *Allobaculum*, and *Bacteroides* were increased in HFD-fed mice supplemented with *Pt*-egg oil. Notably, *unclassified_f_Ruminococcaceae* and *Akkermansia* (belonging to Verrucomicrobia), were enriched by *Pt*-egg oil treatment in obese mice.

**Figure 4:**
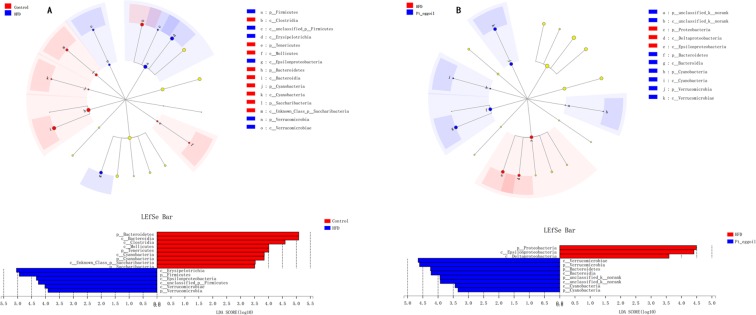
Effects of *Pt*-egg oil on the bacterial taxa in HFD-fed mice (*n* = 4/group). (**A**,**B**) show different taxa between the control and HFD groups, HFD and *Pt*-egg oil groups, respectively.

LEfSe analysis (LDA score log 10 > 4) was conducted to identify specific phylotypes which were changed by *Pt*-egg oil treatment. Firmicutes was increased in HFD mice, mainly including *Erysipelotrichia* and *unclassified_p_Firmicutes* at class level (Fig. 4A). HFD also caused an increase in *Epsilonproteobacteria* at class level, belonging to Proteobacteria (Fig. 4A). HFD feeding decreased the levels of Bacteroidetes, in which *Bacteroidia* was the dominant strain at class level (Fig. 4A). Supplementation with *Pt*-egg oil significantly reduced *Epsilonproteobacteria* abundance, and increased *Bacteroidia* at class level (Fig. 4B), but no significant difference in Firmicutes Phylum.

### *Pt*-egg oil regulated secondary metabolites of gut microbiota

**Figure 5:**
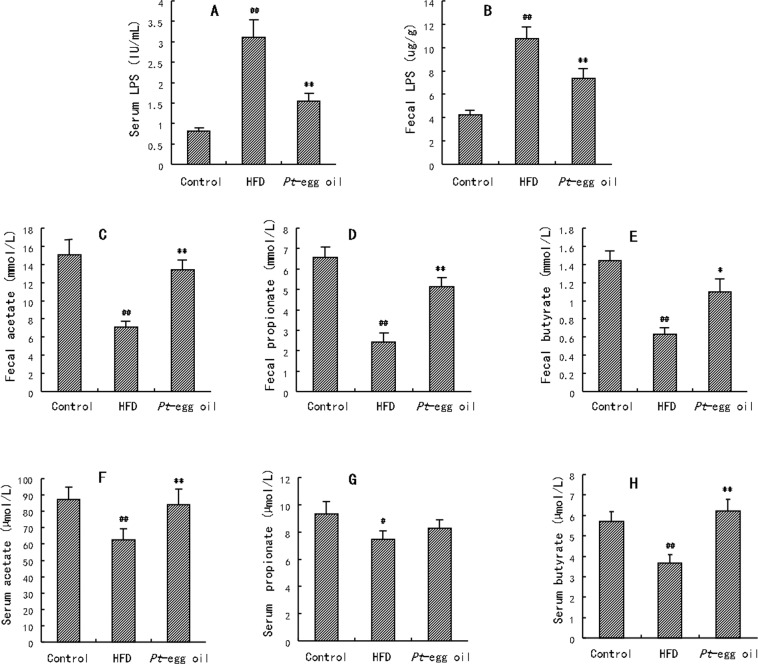
Effects of *Pt*-egg oil on the secondary metabolites of gut microbiota in HFD mice (*n* = 10/group). (**A**) serum LPS concentration; (**B**) fecal LPS concentration; (**C**) fecal acetate concentration; (**D**) fecal propionate concentration; (**E**) fecal butyrate concentration; (**F**) serum acetate concentration; (**G**) serum propionate concentration; (**H**) serum butyrate concentration. ^#^*P* < 0.05, ^##^*P* < 0.01 *vs* control; ^*^P < 0.05, ^**^P < 0.01 *vs* HFD.

Obesity is regulated by gut microbiota through the secondary metabolites, including LPS and SCFAs. As shown in Fig. 5A,B, HFD feeding caused obvious increases in LPS concentrations in serum and in feces (*P*  <  0.01). *Pt*-egg oil significantly decreased serum and fecal LPS concentrations by 50.16% and 31.19%, respectively. Fecal and serum acetate, propionate, and butyrate contents were all remarkably reduced in obese mice compared with control group (Fig. 5C–H, *P*  <  0.01). Interestingly, the three fecal SCFAs were significantly increased in *Pt*-egg oil-receiving mice by 89.56%, 1.13 fold, and 74.60%, respectively. Moreover, Serum acetate and butyrate concentrations were remarkably increased by 39.72% and 69.40% in *Pt*-egg oil group compared with HFD group. However, there is not significant difference in serum propionate level between *Pt*-egg oil group compared with HFD group.

### *Pt*-egg oil down-regulated LPS-dependent pathway and up-regulated SCFAs-dependent pathway

LPS and SCFAs affect obesity through spurring specific cascades, including LPS-dependent TLR4 pathway and SCFAs-dependent GPRs pathway in both adipose and the liver tissues. Table 2 showed that HFD feeding elevated TLR4 and CD14 mRNA relative expression, while *Pt*-egg oil down-regulated the levels of TLR4 and CD14 mRNA in adipose tissues of obese mice (*P*  <  0.05, *P*  <  0.01). Moreover, HFD induced strong decreases in GPR41 and GPR43 mRNA expression in adipose tissues, and *Pt*-egg oil significantly reversed the reductions (*P*  <  0.01). In addition, *Pt*-egg oil markedly lowered TLR4 and CD14 mRNA expression and increased GPR41 and GPR43 mRNA expression in the liver of obese mice (*P*  <  0.05, *P*  <  0.01).

**Table 2:** Effect of *Pt*-egg oil on TLR4, CD14, GPR41, and GPR43 mRNA expression in epididymal adipose and liver tissues of HFD mice.

	Gene	Control	HFD	*Pt*-egg oil
Epididymal adipose tissues	TLR4	1.00 ± 0.11	2.09 ± 0.18^##^	1.44 ± 0.15^*^
	CD14	1.00 ± 0.19	3.06 ± 0.33^##^	1.81 ± 0.20^**^
	GPR41	1.00 ± 0.14	0.35 ± 0.06^##^	0.73 ± 0.09^**^
	GPR43	1.00 ± 0.09	0.42 ± 0.04^##^	0.87 ± 0.08^**^
Liver tissues	TLR4	1.00 ± 0.15	2.47 ± 0.18^##^	1.93 ± 0.15^*^
	CD14	1.00 ± 0.17	2.51 ± 0.24^##^	1.99 ± 0.18^*^
	GPR41	1.00 ± 0.20	0.57 ± 0.05^##^	0.71 ± 0.08^*^
	GPR43	1.00 ± 0.13	0.62 ± 0.06^##^	0.96 ± 0.07^**^

Data are shown as mean ± S.D (*n* = 10/group). Multiple comparisons were done using one way ANOVA. ^##^*P* < 0.01 *vs* control; ^*^P < 0.05, ^**^P < 0.01 *vs* HFD.

## Discussion

Gut microbiota has been considered as an key environmental factor in the development of obesity^15^. In this study, the effects of *Pt*-egg oil on antiobesity and regulation of gut microbiota were investigated. The data showed that *Pt*-egg oil reduced body and adipose weight, serum and hepatic lipids, and epididymal adipose cell size, which suggesting the significant antiobese effects of *Pt*-egg oil. Gut microbiota analysis showed that *Pt*-egg oil prevented the loss of Bacteroidetes and Verrucomicrobia and restrained the increase of Firmicutes, Proteobacteria, and Actinobacteria in obese mice. This study also demonstrated that *Pt*-egg oil mediated LPS and SCFAs production.

Numerous studies have shown that gut microbiota of obese individual is characterized by an abnormal gut microbiota composition^16,17^. We measured community structures of each group by PCA, and it was clear separation between the three group. These suggested that *Pt*-egg oil could help to shape gut microbiota community through natural selection and competing^18^. Some contradictory conclusions on the change of F/B ratio were shown in obese individual. For example, Cui *et al*. reported that an increase F/B ratio developed obesity in HFD mice, and fish and frill oil mixture alleviated the ratio^19^. However, other papers showed a low F/B ratio in obese mice^5,20^. In the present study, HFD feeding elevated F/B ratio in mice, which was restored by *Pt*-egg oil treatment. At the genus level, *Pt*-egg oil reduced *Ruminiclostridium_5*, *Ruminiclostridium*, *Ruminococcaceae_UCG-013*, *Anaerotruncus*, *Oscillibacter*, *Faecalibaculum*, *norank_f_Erysipelotrichaceae*, which are all belonging to Firmicutes, and increased *Prevotellaceae_UCG-001*, *Rikenellaceae_RC9_gut_group*, *Parabacteroides*, and *Paraprevotella*, belonging to Bacteroidetes. Previous studies indicated that Proteobacteria and Actinobacteria were positively correlated with obesity, while Verrucomicrobia was negatively correlated with obesity^21–23^. *Pt*-egg oil treatment reduced Proteobacteria and Actinobacteria, and increased Verrucomicrobia at the Phylum level. Further, *Helicobacter*, belonging to Proteobacteria, *unclasslfied_Coriobacteriaceae* and *Coriobacteriaceae_UCG-002*, belonging to Actinobacteria, were lowered by *Pt*-egg oil. The numbers of *unclassified_f_Ruminococcaceae* and *Akkermansia*, belonging to the Phylum Verrucomicrobia, were also elevated in obese mice when treated with *Pt*-egg oil. Noticeably, the Gram-negative Desulfovibrio genus was significantly reduced in *Pt*-egg oil-treated mice, which is responsible for inflammation and obesity resulting from lipid A structures of LPS^24^. *Lactobacillus* and *Bacteroides*, the beneficial intestinal bacteria, were also promoted by *Pt*-egg oil, which is proved to positively relate to intestinal integrity, glucose tolerance or attenuated obesity^25,26^. In addition, *Pt*-egg oil reversed HFD-decreased *Bacteroidia* at class level, which is negatively correlated with obesity. Meanwhile, *Erysipelotrichia* and *unclassified_p_Firmicutes*, belonging to Firmicutes, was reduced by *Pt*-egg oil, which could positively stimulate obesity and hyperlipemia^27^. Similar results can also be found in studies on other marine bioactive lipids^28,29^. These indicate that *Pt*-egg oil can alleviate obesity by directly modulating gut microbiota.

Special microbiota can produce SCFAs, including *Bacteroides*, *Lactobacillus*, *Bifidobacterium*, *Prevotella*, *Lachnospiraceae*, *Butyricimonas*, *Alloprevotella*, *Clostridium*, *Allobaculum* etc.^30–32^. Our results showed that *Pt*-egg oil promoted the abundance of the SCFAs-producing microbiota *Lachnospiraceae_NK4A136_group*, *norank_f_Lachnospiraceae*, *Prevotellaceae_UCG-001*, *Ruminiclostridium_9*, *Butyricimonas*, *Alloprevotella*, *Clostridium_sensu_stricto_1*, *Allobaculum*, and *Bacteroides*, but lowered *Bifidobacterium*. After transporting into blood, SCFAs can be taken up by body tissues and subsequently act as substrates and signal melecules^33^. Acetate could promote cholesterol synthesis, and propionate and butyrate could modulate lipid/cholesterol metabolisms^34^. *Pt*-egg oil significantly enhanced fecal acetate, propionate, and butyrate contents in obese mice, and also increased serum acetate and butyrate concentrations. These changes may be associated with the improvement in many factors in *Pt*-egg oil-treated mice, such as regulation of gut microbiota composition, decrease in body weight gain, and others^35^. As SCFAs receptors, GPR41 and GPR43 take part in such metabolic pathways, including lipolysis and lipogenesis^36^. Many studies proved that the increases in GPR41 and GPR43 expression could mitigate serum lipids and obesity^37,38^. In this study, *Pt*-egg oil increased SCFAs and GPR41 and GPR43 mRNA expression. *Pt*-egg oil-treated mice also showed significant improvement on serum and hepatic lipids levels, body weight gain, adipocyte size, and adipocytokines. These demonstrate that *Pt*-egg oil-inducted SCFAs generation by regulating special gut flora positively contributes to antiobese effects in mice.

LPS can provoke obesity, inflammation, and even diabetes with the most potent capability^39^. Significantly, the abundances of Proteobacteria Phylum, *Desulfovibrio* and *Enterorhabdus*, LPS producing bacteria, were reduced by *Pt*-egg oil treatment. And this regulation was accompanied with decreases in serum and fecal LPS concentrations. Thus, we suggest that the inhibition of pathogenic LPS-producing bacteria by *Pt*-egg oil might result in a decrease of the decrease of the LPS load into the systemic circulation, and may revealed that the antiobese effects of *Pt*-egg oil is, in part, responsible for the dramatically reduction in LPS. Previous study has shown that LPS could induce intestinal barrier integrity impared^40^. *Pt*-egg oil stimulated the elevations in intestinal barrier protectors abundance, such as *Lachnospiraceae_NK4A136_group* and *norank_f_Lachnospiraceae*^41^. In addition, LPS reduction has been repeatedly shown to improve obesity and obesity-related cytokines^42^, and in this study, *Pt*-egg oil decreased LPS concentrations which were associated with enhanced adiponectin, and lowered leptin, resistin, and TNF-α. TLR4/CD14 pathway triggered by LPS is the primary mechanism linking gut bacteria to alleviate obesity^43^. In HFD-fed TLR4-deficient mice, the epididymal adipose weight and blood LPS level were only 69% and 18% of HFD mice, respectively^44^. In the present study, *Pt*-egg oil inhibited the elevation of TLR4 and CD14 gene mRNA expression in adipose and liver tissues, which is conjuncted with the regulation of gut microbiota, declines in LPS levels, and decreases in body and fat weight. All of these demonstrate that *Pt*-egg oil alleviates obesity by improving gut microbiota and LPS.

In summary, this paper demonstrated that *Pt*-egg oil alleviated HFD-induced obesity and improved lipids metabolism in mice. These were directly related with the modulation of gut microbiota community. *Pt*-egg oil-regulated specific bacteria could improve body weight and lipids metabolism by down-regulation of LPS/TLR4 pathway and up-regulation of SCFAs/GPRs signaling. In short, it suggested that *Pt*-egg oil may be an alternative food supplement in alleviating obesity and improving other intestinal diseases.

## Materials and methods

### Statement

All methods in these experiments were conducted according to the relevant guidelines and regulations of Qingdao University. Moreover, the animals’ experimental protocols were approved by the ethical committee for experimental animal care at Qingdao University.

### Preparation of *Pt*-egg oil

Tongqu Aquatic Food Company (Zhoushan, Zhejiang, China) provided *Portunus trituberculatus* eggs. *Pt*-egg oil was prepared according to our previous study^14^. *Pt*-egg oil contained 52.05% phospholipids, 8.61% free fatty acids (containing 23.84% saturated fatty acids and 76.16% unsaturated fatty acids, especially 33.23% eicosapentaenoic acid and docosahexaenoic acid), 32.38% triglyceride, 4.79% total cholesterol, and 971.79 μg/g astaxanthin^14^.

### Animal experiments

C57BL/6J mice (male, 16-18 g, 4-5 weeks) were from Vital River Laboratory Animal Center (Beijing, China; licensed ID SCXK2014-0004). They were fed in a 12:12 h light-dark condition at 22–24 °C in normal cages. The mice were randomized into three groups (10 per group): control group (fed with normal chow diet: 74% corn starch, 16% casein, and 10% corn oil based on weight, 4.09 kcal/g), high fat diet (HFD)-feeding group (fed with HFD: 29% corn starch, 16% casein, 10% corn oil, and 45% lard based on weight, 6.57 kcal/g), and *Pt*-egg oil group (administrated with HFD and 600 mg/kg *Pt*-egg oil intragastrically). After 16 weeks treatment, faeces were collected form each mouse feeding in metabolism cages. The animals were sacrificed after 12 h fasting and serum was collected to measure serum lipids and adipokines contents. The liver and epididymal adipose tissues were separated rapidly for hematoxylin and eosin (H&E) stain or measurement of hepatic lipids.

### Serum and hepatic lipids analysis

Serum TC, TG, HDL-c, and LDL-c concentrations were detect by commercial kits (Jiancheng, Najing, Jiangsu, China).

Hepatic TG and TC levels measured by the same kits as used in serum analysis after the lipids in liver extracted by the method of Folch *et al*.

### Adipokines detection

Serum adiponectin, resistin, leptin, and tumor necrosis factor-α (TNF-α) levels were detected by the ELISA kits (Invitrogen, Carlsbad, CA, USA) according to the manufacture’s instructions.

### H&E stain

H&E stainning was performed in epididymal adipose tissues after formalin, paraffin embedded, sectioned. Fat microscopic structures were photographed by a fluorescence microscope (Eclipse Ci, Nikon, Japan) and using CaseViewer 2.0 to get adipose cell size. The size in control group was defined as 1.

### Serum and fecal LPS measurement

After diluted to 20% (v/v) with water, serum was heated to 70 °C to inactivate proteins. Serum LPS was measured by ELISA kit (Invitrogen, Carlsbad, CA, USA).

Faeces was homogenized in ice-cold Millipore H_2_O, and then centrifuged at 7,500 × g for 15 min. The supernatant fraction was heated to 70 °C to inactivate proteins. Fecal LPS was measured according to the aforementioned methods.

### Serum and fecal SCFAs determination

Serum and fecal SCFAs levels were evaluated according to our previous study^45^.

### Fecal DNA extraction

DNA (*n* = 4 per group) was extracted from feces by QIAamp DNA Stool Mini Kit (Qiagen, Dusseldorf, Germany).

### Intestinal microflora analysis

PCR amplify, sequences analysis, taxonomic identification, alpha and beta diversities were all performed according to our previous stidies^45,46^.

Quantitative real time polymerase chain reaction (qRT-PCR) analysis. Total epididymal adipose and hepatic mRNA was isolated using a TRIzol reagent, and then reverse transcribed into cDNA. The expression levels of TLR4, CD14, GPR41, and GPR43 were analyzed as our previous study.46 β-actin was used as the control and mean expression level in control group was set as 1. The primers were as follow: TLR4 (F, 5′-TCAGAGCCGTTGGTGTATCTT-3′, R, 3′-AACTCTTCAGGGACGACTCC-5′), CD14 (F, 5′-TTGGCTTGTTGCTGTTGCTTC-3′, R,3′-TAGAGTTGTAGAACTTGGAGGCG-5′), GPR41 (F, 5′-CTGCTCCTGCTCCTCCTC-3′, R, 3′-CCAGGCGACTGTAGCAGT-5′), GPR43 (F, 5′-TTCTTACTGGGCTCCCTGCC-3′, R, 3′-TACCAGCGGAAGTTGGATGC-5′), and β-actin (F, 5′-CAAGGCATTGCTGACAGGATG-3′, R,3′-GGTCGTCTACACCTAGTCGT-5′).

### Statistical analysis

OTUs univariate differential value at Phylum level was tested according to Fisher’s test. *P*  <  0.05 is considered significance after Benjamini-Hochberg method correcting. Data are shown as mean ± S.D and statistically analyzed by SPSS 17.0 software (SPSS Inc., Chicago, IL, USA). Difference between three groups is conducted by Student’s test and *P*  <  0.05 is considered significance.
